# The Medical Society of the County of Erie

**Published:** 1893-02

**Authors:** 


BUFFALO MEDICAL AND SURGICAL JOURNAL

A MONTHLY REVIEW OF MEDICINE AND SURGERY.

*EDITORS:*

THOMAS LOTHROP, M. D. - - WM. WARREN POTTER, M. D.

All communications, whether of a literary or business character, should be addressed to the managing editor: 284 Franklin Street, Buffalo, N. Y.

Vol. XXXII. FEBRUARY, 1893. No. 7.

### THE MEDICAL SOCIETY OF THE COUNTY OF ERIE.

This society held its annual meeting January 10, 1893, an account of which will be found under the head of Society Proceedings. Though the day of the meeting was the coldest thus far of the year, it was a large gathering of interested professional men, who dispatched the work of the society with promptness and apparent satisfaction to all.

The President, in his address, dealt with some of the essential needs of the society, and we hope every member will read the address, which is published in another column of this issue. It contains suggestions on many subjects that require careful consideration, and some action ought to be taken with reference to some of them at an early day. We invite attention, particularly, to what he has said regarding the State Medical Examining and Licensing Board. It is essential that every physician should become familiar with the requirements of the law regarding license to practise medicine, so that he may be enabled to point out the essentials to those who may become applicants for license under the statute.

There has been great danger that this beneficent law would be rendered nugatory by tampering with it through unauthorized individual efforts. We say unauthorized, because, in reality, no amendment to the law should be made without the recommendation of the Examining Boards and the Regents of the University, which should be considered the authorized source whence all amendments should spring. These are the official executors of the law, and know wherein it may need modification or amendment. There should not only be a firm resolution on the part of the profession of medicine throughout the State to prevent any weakening of its force and effect, but also a standing together for the purpose of making it better and stronger. Only recently an attempt was made on the part of Niagara University to widen the doors with reference to preliminary educational qualifications of students, and a bill looking to a modification of the present requirements was introduced in the legislature by Dr. Goldberg, Assemblyman from the second district, who is also one of the members of this society. This, at first, seemed bad for both the instigators of the bill and the honorable member of the legislature who introduced it, but upon further explanation and understanding, it has been disovered that neither is Dr. Goldberg in favor of the bill, nor will the originators of it further urge its passage, all concerned having been convinced that it would be unwise to modify the present law in the direction named.

The Buffalo *Enquirer*, of January 14th, contained a statement from Dr. Goldberg, to the effect that he would not move the bill from its present place unless instructed to do so by the Medical Society of the State of New York. This seems a satisfactory adjustment of the matter, and all parties interested are apparently satisfied. This, however, only shows how watchful the friends of medical education must be to prevent unwise adverse legislation on the subject.

The scientific part of the meeting was most interesting, and the attendance was large even to the late hour of adjournment. An account of these proceedings in part will be published in a future number of the JOURNAL, which will contain the papers and discussion on The Relation of Gonorrhea to Pelvic Disease in Women.

